# Nrf2 regulates the arginase 1^+^ microglia phenotype through the initiation of TREM2 transcription, ameliorating depression-like behavior in mice

**DOI:** 10.1038/s41398-022-02227-y

**Published:** 2022-10-31

**Authors:** Lujuan He, Yi Zheng, Lixuan Huang, Jingyi Ye, Yinyi Ye, Hanyue Luo, Xi Chen, Wei Yao, Jiaxu Chen, Ji-chun Zhang

**Affiliations:** 1 Department of Physiology, School of Medicine, Jinan University, Guangzhou, 510632 China; 2 Department of Anesthesiology, Guangzhou Women and Children’s Medical Center, Guangzhou Medical University, Guangzhou, China; 3 Guangzhou Key Laboratory of Formula-Pattern of Traditional Chinese Medicine, School of Traditional Chinese Medicine, Jinan University, Guangzhou, 510632 China

**Keywords:** Molecular neuroscience, Depression

## Abstract

The expression of the triggering receptor on myeloid cell-2 (TREM2) knockdown in microglia from the lateral habenula (LHb) reportedly induces depression-like behaviors in mice. However, the key molecular mechanism that mediates major depressive disorder (MDD) pathogenesis remains elusive. We herein show that Nrf2 regulates TREM2 transcription and effects TREM2 mRNA and protein expression. The activation of Nrf2 by sulforaphane (Nrf2 activator) increases the microglial arginase 1^+^ phenotype by initiating TREM2 transcription in the medial prefrontal cortex (mPFC) and ameliorates depression-like behavior in CSDS mice. The knockout of Nrf2 decreases TREM2 and the microglial arginase 1^+^ phenotype in the mPFC of Nrf2 KO mice with depression-like behavior. Downregulating TREM2 expression decreases the microglial arginase 1^+^ phenotype in the mPFC, resulting in depression-like behavior in SFN-treated CSDS mice. Finally, the knockout of Nrf2 and downregulation of TREM2 expression decreases the microglial arginase 1^+^ phenotype in the mPFC of Nrf2 KO mice and SFN-treated CSDS mice were associated with the brain-derived neurotrophic factor (BDNF)-tropomyosin receptor kinase B (TrkB) signaling pathway. These data indicate that alterations in the interaction between Nrf2 and TREM2 may play a role in the pathophysiology of depression-like behavior in mice.

## Introduction

Microglia are immune cells in the central nervous system that play an important role in maintaining normal brain function [1]. Accumulating evidence suggests that microglia are associated with various neuropsychiatric disorders, including depression, through their release of inflammatory cytokines, regulation of cell apoptosis via phagocytosis, and synaptic plasticity [2, 3]. Further, microglia can adopt proinflammatory and anti-inflammatory phenotypes according to their microenvironment [4–6]. Activation of the anti-inflammatory phenotype regulates the release of various anti-inflammatory factors, which lead to neuronal protection [4, 7–9]. Triggering receptor expressed on myeloid cell-2 (TREM2) is a cell surface receptor the belongs to the lectin-like immunoglobulin superfamily. Expressed at higher levels in microglia than neurons, TREM2 functions as a key regulator of the inflammatory response [10]. Several lines of evidence suggest that TREM2 plays an essential role in the modulation of microglial functions, including phagocytosis and the production of proinflammatory cytokines [11]. TREM2 knockdown amplifies tumor necrosis factor-α (TNF-α) and NO synthase-2 transcription (NOS2) expression in microglia, while TREM2 overexpression significantly reduces TNF-α, interleukin-1β (IL-1β) and NOS2 expression [12]. In humans, TREM2 plays an essential role in maintaining immune homeostasis in the brain by promoting the clearance of tissue debris and resolution of latent inflammatory responses [13]. Comparing the postmortem prefrontal cortex of patients with bipolar disorder (BD) with those of healthy controls, one study found that TREM2 is relatively downregulated in the dorsolateral prefrontal cortices (DLPFCs) of patients with BD [14]. In addition, a murine investigation reported that specific TREM2 knockdown in microglia from the lateral habenula (LHb) induces proinflammatory cytokine expression and microglial activation, resulting in depressive-like behaviors [15]. However, insight into the expression and biological functions of TREM2 in the context of major depressive disorder (MDD) remains lacking.

Nuclear factor erythroid 2-related factor 2 (Nrf2) is a key transcription factor that regulates antioxidant and anti-inflammatory responses by promoting the transcription of phase II detoxification enzymes [16–21]. An isothiocyanate compound derived from broccoli, sulforaphane (SFN: 1-isothiocyanato-4-methylsulfinylbutane) is a potent activator of Nrf2 that exerts antioxidant and anti-inflammatory effects mediated by the activation of the Nrf2 signaling pathway [22–25]. Accumulating evidence suggests that Nrf2 is crucial in the pathogenesis of depression [26, 27]. Recently, our study revealed that Nrf2 activates brain-derived neurotrophic factor (BDNF) and thus contributes to antidepressant-like effects on rodents [28]. Further, the activation of Nrf2 by SFN (Nrf2 activator) confers stress resilience through the activation of anti-inflammatory microglia, which are associated with BDNF upregulation [29]. Such findings suggest that Nrf2 may regulate the microglial anti-inflammatory phenotype through the initiation of TREM2 transcription and thus ameliorate depression-like behavior in mice.

The present study reports that Nrf2 regulates TREM2 transcription, inducing TREM2 mRNA and protein expression. The activation of Nrf2 by SFN increased the microglial arginase 1^+^ phenotype by initiating TREM2 transcription in the medial prefrontal cortex (mPFC) and thus ameliorated depression-like behavior in CSDS mice. The knockout of Nrf2 or downregulation of TREM2 expression decreased the microglial arginase 1^+^ phenotype in the mPFC of Nrf2 KO mice and SFN-treated CSDS mice. Finally, we identified an association between these effects and the BDNF-tropomyosin receptor kinase B (TrkB) signaling pathway.

## Materials and methods

### Animals and cell culture

Male adult C57BL/6 mice (8 weeks old, 20–25 g, Guangdong Experimental Animal Center), CD1 mice (14 weeks old, 40–45 g, Guangdong Experimental Animal Center), male adult Thy1-yellow fluorescent protein (YFP) mice, and Nrf2 KO mice (The Jackson Laboratory, Bar Harbor, ME) were used in the experiments. The animals were housed at a controlled temperature on a 12-h light/dark cycle (lights on between 07:00 and 19:00) with ad libitum access to food and water. The study protocol was approved by the Institutional Animal Care and Use Committee of Jinan University. All experiments were performed in accordance with the Guide for Animal Experimentation of Jinan University. Each genotype age-matched animal was randomly assigned to the experimental group. The sample size was based on previous experimental designs [28]. Batches of mice were tested independently and pooled together for the final analysis. As a result, the sizes of the groups are not uniform. These criteria are not predetermined.

BV2 microglial cells were cultured in high-glucose Dulbecco’s modified Eagle’s medium supplemented with 10% fetal bovine serum (Excell Bio., Taicang, China) and penicillin (100 units/mL)-streptomycin (100 μg/mL, HyClone). The cells were incubated at 37 °C in a humidified incubator containing 5% CO_2_. The BV2 cell lines were tested for mycoplasma contamination.

For the primary culture of microglia, glia was prepared for the experiments within 24 h of newborn C57BL/6 mice. Whole brains were removed after disinfection of the scalp with 75% alcohol. The whole brains were placed in 4 °C precooled sterile D-Hank’s buffer, slightly cleared of blood cells, and then transferred to petri dishes containing precooled D-Hank’s buffer. The meninges and blood vessels on the brain surface were stripped under a microscope, and the brain tissue was transferred to an ice centrifuge tube. Trypsin (0.25%) was added for digestion at room temperature for 10 min, and the digestion was stopped with complete DMEM/F-12 medium. After the brain tissue was fully dissociated, the supernatant in the centrifuge tube was passed through a 100 μm filter, the filtrate was centrifuged at 1500 RPM for 10 min at room temperature, the supernatant was absorbed and discarded, and the cells were resuspended in DMEM/F-12 medium. After thorough resuspension, cells were seeded in a T75 culture flask coated with a cell adhesive at a density of 3 × 10^6^ cells/mL and placed in an incubator at 37 °C with 5% CO_2_ for culture. The medium was changed every 3 days. After approximately 10‒12 days of being cultured, the astrocytes were dissociated for 30 min with 0.25% trypsin diluted in DMEM at a dilution ratio of 1:4. The microglia were then plated in culture plates for use [30].

The mouse pcDNA3.1-*Nrf2* plasmid used for cell transfection was gifted by Dr. Masayuki Yamamoto (Tohoku University Graduate School of Medicine, Sendai, Japan), the mouse mGST-*Trem2* plasmid by Dr. Zhentao Zhang (Department of Neurology, Renmin Hospital of Wuhan University, Wuhan, China), the human full-length *Trem2* promoter plasmid by Dr. Marek Rusin (Maria Skłodowska–Curie Institute-Oncology Center, Gliwice, Poland). The site 1, 2, and 3 WT and mutant *Trem2* promoter luciferase plasmids were constructed by TsingKe Biological Technology (Wuhan, China). HEK293T or BV2 cells were transfected using Lipofectamine 3000 (Invitrogen) for 24 h according to the manufacturer’s instructions.

### SFN, LPS, and TREM2-HDO treatments

SFN (1 μM for primary microglia or BV2 cells; LKT Laboratories, Inc., St Paul, MN, USA) was dissolved in dimethylsulfoxide. Lipopolysaccharide (LPS, 1 μg/mL for primary microglia or BV2 cells; L-4130, serotype 0111: B4, Sigma-Aldrich, St. Louis, MO, USA) was dissolved in physiological saline. SFN (10 mg/kg in physiological saline containing 10% corn oil.) was injected intraperitoneally (i.p.) as described in previous reports by a researcher blinded to the treatment [31–33]. The selection of doses of SFN (1 μM) and LPS (1 μg/mL) appropriate for cell cultures was informed by a previous study [10, 28].

The antisense oligonucleotides (ASOs) and cRNA targeting *Trem2* were purchased from TsingKe Biological Technology (Wuhan, China) and solubilized in 0.9% sterile saline immediately before use. For the generation of TREM2-DNA/RNA heteroduplex oligonucleotides (HDOs), equimolar amounts of DNA and cRNA strands were heated in 0.9% sterile saline at 95 °C for 5 min and slowly cooled to room temperature. TREM2-HDO carried locked nucleic acids (LNAs) at each end flanking the central base of DNA with or without the CY5 label and carried 2′-O-methyl at each end flanking the central base of cRNA. The sequences of ASOs and cRNA targeting *Trem2* used in our experiments are listed below. TREM2-ASO: G(L)^C(L)^C^C^A^G^C^A^T^C^T^T^G^G^C^C^A^C(L)^A(L)^G(L); TREM2-cRNA: c(M)^u(M)^g(M)^uggccaagaugcuggg(M)^c(M). L represents locked nucleic acids; M, 2′-O-methyl modifications; and ^, a phosphorothioate bond. Other reagents were purchased commercially.

### Intracerebroventricular (i.c.v.) injection

For the i.c.v. injection of TREM2-HDO, mice were anesthetized with 1% pentobarbital sodium and fixed to the stereotaxic apparatus. TREM2-HDO (200 nM in 2 μl) was injected into the right lateral ventricle at the following stereotaxic coordinates: 0.8 mm lateral, 2.1 mm ventral, and 0.74 mm from the bregma. The needle remained in place for 5 min after the injection of the drugs, followed by slow removal. The mice were placed on a heating pad until they recovered from the anesthesia.

### Chronic social defeat stress (CSDS) model, social interaction test (SIT), locomotion, forced swimming test (FST), 1% sucrose preference test (SPT), luciferase assay, chromatin immunoprecipitation (ChIP) assay, quantitative PCR (qPCR) assay, immunoblotting assay, immunofluorescence staining, and dendritic spine analysis

See the supplemental information for detailed descriptions of the CSDS mode, SIT, locomotion, FST, 1% SPT, luciferase reporter assay, ChIP assay, qPCR assay, immunoblotting assay, immunofluorescence staining, and dendritic spine analysis.

### Statistical analysis

All results are presented as the means ± standard errors of the means (SEMs). All data were analyzed using PASW Statistics 20 software. Differences between the groups were evaluated using a one-way analysis of variance (ANOVA) followed by the post hoc Fisher least significant difference (LSD) test. Student’s *t* test was used to compare the differences between two groups. *P* values of <0.05 were considered significant.

## Results

### Nrf2 regulates TREM2 transcription

**Fig. 1:**
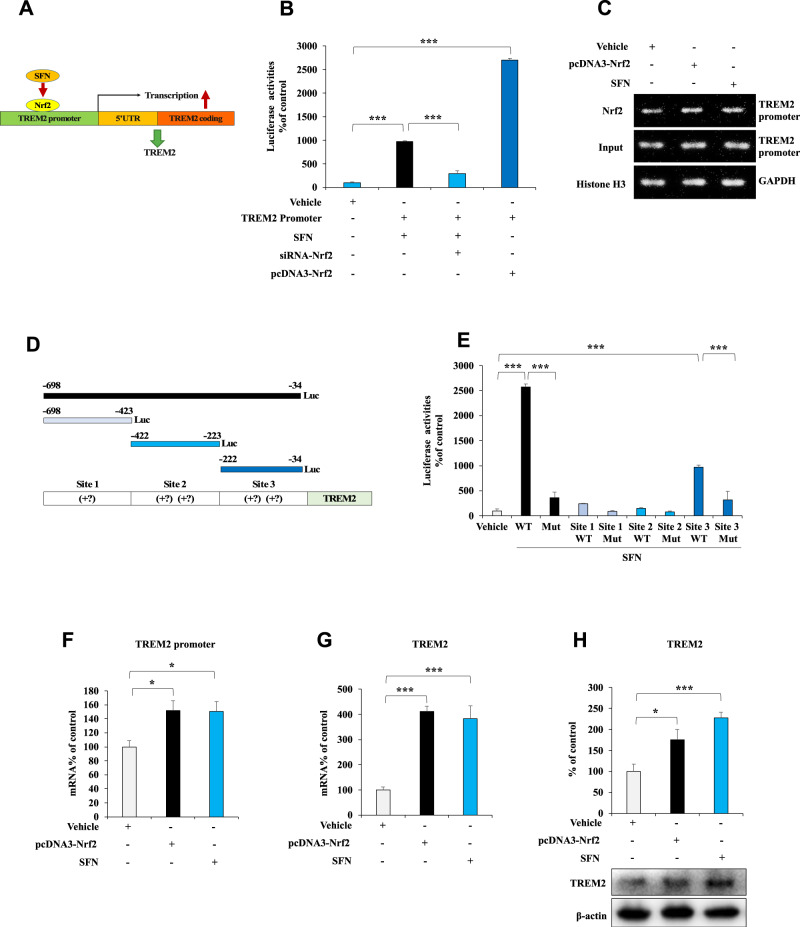
Nrf2 regulates TREM2 transcription. **A** Graphical illustration of the mechanism by which Nrf2 regulates transcription. **B** TREM2 promoter activity in BV2 cells treated with SFN or overexpressing Nrf2 and/or siRNA-Nrf2. The data are presented as the means ± SEMs (*n* = 4). ***P < 0.001 (one-way ANOVA). **C** ChIP-PCR assay of the TREM2 promoter. Nrf2 protein–DNA cross-linked samples were obtained from BV2 cells treated with vehicle or SFN or overexpressing Nrf2 via coimmunoprecipitation with an anti-Nrf2 antibody. PCR was conducted using TREM2 promoter primers. **D** Graphical illustration of the TREM2 promoter. **E** Activity of different lengths of the TREM2 promoter in BV2 cells treated with SFN. The data are presented as the means ± SEMs (*n* = 4). ***P < 0.001 (one-way ANOVA). **F**–**H** qPCR analysis and Western blot assays of TREM2 promoter, TREM2 mRNA expression, and TREM2 protein expression in BV2 cells after vehicle treatment, SFN treatment, or Nrf2 overexpression. The data are presented as the means ± SEMs (*n* = 4 or 5). *P < 0.05 and ***P < 0.001 (one-way ANOVA).

We tested the hypothesis that Nrf2 regulates TREM2 transcription by analyzing the DNA sequences of the promoter regions in the human TREM2 gene. We found numerous potential Nrf2 consensus binding motifs. A luciferase assay with BV2 cell lysates from SFN-treated cells or cells cotransfected with Nrf2 and the human TREM2 promoter constructs showed that the human TREM2 promoter contains a major binding site for Nrf2 (Fig. 1A, B). We performed a ChIP assay with cells treated with SFN or those overexpressing Nrf2 using an Nrf2-specific antibody to explore whether Nrf2 indeed binds to the TREM2 promoter. The PCR analysis of genomic DNA immunoprecipitated with a Nrf2 antibody revealed the Nrf2 interaction with the TREM2 promoter was more enhanced by SFN treatment or Nrf2 overexpression enhanced than by the vehicle (Fig. 1C). We prepared a series of luciferase-conjugated constructs with systemic truncation of the Nrf2 binding sites in the human TREM2 promoter to further analyze the Nrf2 binding site for the human TREM2 promoter (Fig. S1). The luciferase assay results showed that full-length constructs and site 3 (from −222 to −34) displayed luciferase activities, whereas mutation of this motif completely abolished promoter activity (Fig. 1D, E). These findings may implicate site 3 as the major binding motif for Nrf2. We also analyzed the mouse TREM2 promoter and found the same sequence as the human TREM2 promoter at site 3 containing the Nrf2 binding site (Fig. S2).

Next, we conducted quantitative PCR (qPCR) assays and immunoblotting with BV2 cells treated with SFN and those overexpressing Nrf2 to assess whether Nrf2 mediates TREM2 mRNA and protein expression. The qPCR analysis showed that SFN treatment and Nrf2 overexpression increased the activity of the TREM2 promoter, as well as TREM2 mRNA levels (Fig. 1F, G). In addition, immunoblotting revealed that SFN treatment and Nrf2 overexpression upregulated TREM2 expression in BV2 cells (Fig. 1H). These data support the hypothesis that Nrf2 modulates TREM2 mRNA and protein expression.

### TREM2 can also influence Nrf2 expression

We used a DNA/RNA heteroduplex oligonucleotide (HDO), a technological product used for gene silencing, to downregulate TREM2 expression in BV2 cells and assess whether TREM2 also influences Nrf2 expression [34, 35]. Immunoblotting and qPCR showed that TREM2-HDO decreased TREM2 and Nrf2 mRNA and protein expression in a dose-dependent manner (Fig. S3A, B). Further, we also examined TREM2 and Nrf2 mRNA and protein levels in GST-TREM2-treated BV2 cells. Immunoblotting and qPCR showed that GST-TREM2 increased TREM2 and Nrf2 mRNA and protein expression in a dose-dependent manner (Fig. S3C, D). These results suggest that TREM2 also influences Nrf2 expression.

### Activation of Nrf2 by SFN induces an anti-inflammatory microglial phenotype through increased TREM2 expression in LPS-treated primary microglia

**Fig. 2:**
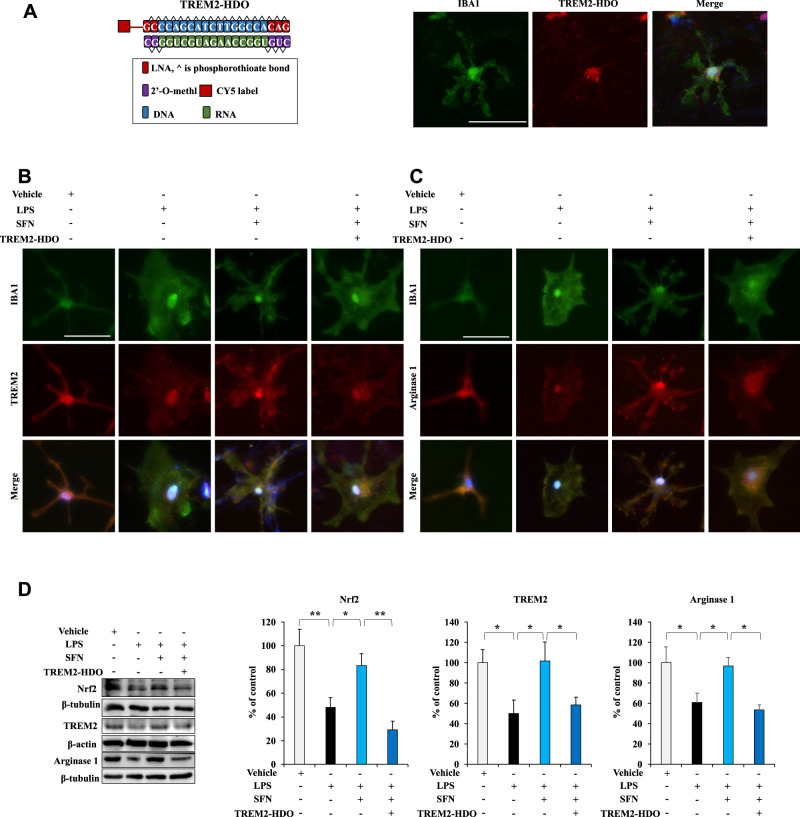
Activation of Nrf2 by SFN induces an anti-inflammatory microglial phenotype according to TREM2 expression in LPS-treated primary microglia. **A** Schematic illustration of the construction of TREM2-HDO. The internalization of CY5-TREM2-HDO visualized by microscopy at 24 h following transfection of CY5-TREM2-HDO (200 nM), Scale bar = 50 μm. **B** Representative images of TREM2 and IBA1 staining in primary microglia. Scale bar = 50 μm. **C** Representative images of arginase 1 and IBA1 staining in primary microglia. Scale bar = 50 μm. **D** Western blot assay showing Nrf2, TREM2, and arginase 1 protein expression in primary microglia. The data are presented as the means ± SEMs (*n* = 5 or 6). **P < 0.01 and ***P < 0.001 (one-way ANOVA).

Our results suggest that Nrf2 regulates TREM2 transcription, resulting in TREM2 mRNA and protein expression. To explore whether the activation of Nrf2 by SFN can induce an anti-inflammatory microglial phenotype through increased TREM2 expression in LPS-treated primary microglia, we cultured primary microglia and used TREM2-HDO to downregulate TREM2 expression. CY5-TREM2-HDO was absorbed in primary microglia after treatment for 24 h (Fig. 2A). Immunofluorescence staining showed the colocalization of TREM2 with ionized calcium binding adaptor molecule 1 (IBA1) and arginase 1 with IBA1 in primary microglia (Fig. 2B, C). LPS decreased TREM2 and arginase 1 immunoreactivity and increased IBA1 immunoreactivity in primary microglia; these effects were reversed with SFN treatment (Fig. 2B, C). Further, TREM2-HDO attenuated the increased TREM2 and arginase 1 immunoreactivity, as well as the decreased IBA1 immunoreactivity, in LPS + SFN-treated primary microglia (Fig. 2B, C). Immunoblotting showed that LPS decreased Nrf2, TREM2, and arginase 1 expression in primary microglia; these effects were also reversed by SFN treatment (Fig. 2D). TREM2-HDO attenuated the increased Nrf2, TREM2, and arginase 1 expression in LPS + SFN-treated primary microglia (Fig. 2D). Arginase 1 is a marker of the anti-inflammatory phenotype of microglia [4–7]. Consequently, activation of Nrf2 by SFN induces an anti-inflammatory microglial phenotype through increased TREM2 expression in LPS-treated primary microglia.

### Nrf2 and TREM2 levels and the microglial arginase 1^+^ phenotype in the medial prefrontal cortex (mPFC) are involved in depression-like behavior in CSDS mice

**Fig. 3:**
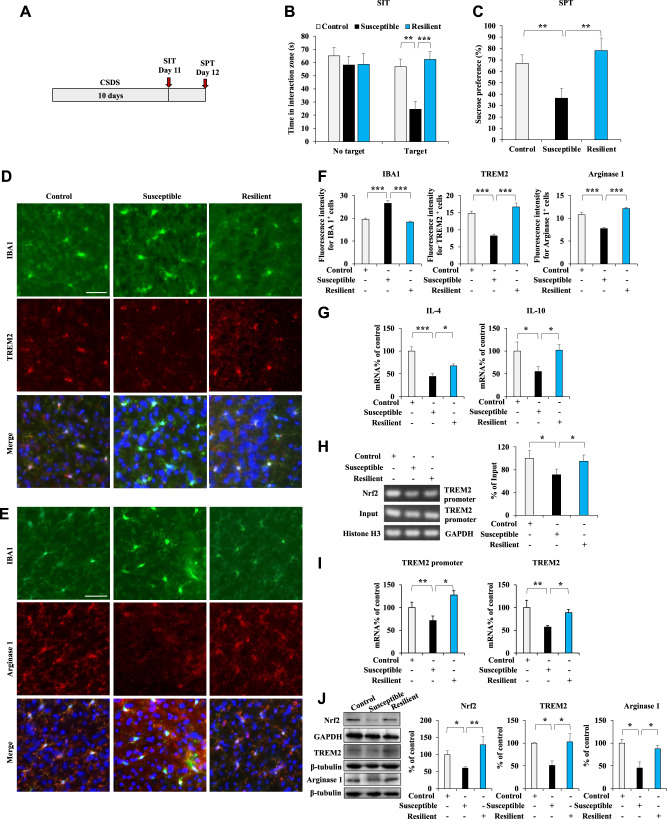
Nrf2 and TREM2 protein levels and the microglial arginase 1^+^ phenotype in the mPFC are involved in depression-like behavior in CSDS mice. **A** Schedule of behavior tests and treatment. **B** The social interaction test for no-target and target time (mean ± SEM, *n* = 8 or 9 per group, one-way ANOVA, **p < 0.01, ***p < 0.001). **C** The sucrose preference test (mean ± SEM, *n* = 8 or 9 per group, one-way ANOVA, **p < 0.01). **D**–**F** Representative images and quantification of TREM2, IBA1, and arginase 1 staining in the mPFC. Scale bar = 50 μm (mean ± SEM, *n* = 5 per group, one-way ANOVA, ***p < 0.001). **G** qPCR assay for IL-4 and IL-10 in the mPFC. The data are shown as the mean ± SEM (*n* = 5 or 6). *P < 0.05 and ***P < 0.001 (one-way ANOVA). **H** ChIP-PCR assay of the TREM2 promoter. Nrf2 protein–DNA cross-linked samples were obtained from the mPFC of control, susceptible, and resistant mice via coimmunoprecipitation with an anti-Nrf2 antibody. PCR was conducted using TREM2 promoter primers. The data are presented as the mean ± SEM (*n* = 5). *P < 0.05 (one-way ANOVA). **I** qPCR assay for the TREM2 promoter and TREM2 in the mPFC. The data are shown as the mean ± SEM (*n* = 5 or 6). *P < 0.05 and **P < 0.01 (one-way ANOVA). **J** Western blot assay for Nrf2, TREM2, and arginase 1 protein expression in the mPFC. The data are presented as the mean ± SEM (*n* = 4 or 6). *P < 0.05 and **P < 0.01 (one-way ANOVA).

The in vitro study showed that Nrf2 regulates TREM2 transcription and that activation of Nrf2 by SFN induces the microglial arginase 1^+^ phenotype through increased TREM2 expression in LPS-treated primary microglia. We continued to investigate the roles of Nrf2 and TREM2 levels and the microglial arginase 1^+^ phenotype in the mPFC in the context of depression-like behavior of CSDS mice. After 10 days of CSDS, the mice were subjected to social interaction test and 1% sucrose preference test (SPT) to examine how susceptible or resilient the mice were to CSDS (Fig. 3A–C). Immunofluorescence staining with anti-TREM2 and anti-IBA1 antibodies in the mPFC sections revealed decreased TREM2 immunoreactivity and increased IBA1 immunoreactivity in the susceptible mice but not in the resilient mice (Fig. 3D–F). In addition, our data showed that CSDS led to a decrease in arginase 1^+^ microglial immunoreactivity in the mPFC of susceptible mice but not of resilient mice (Fig. 3E, F). The qPCR revealed decreased anti-inflammatory cytokine (IL-4 and IL-10) expression in the mPFC of susceptible mice and the opposite in resilient mice (Fig. 3G). Notably, the ChIP-PCR results showed that Nrf2 partially dissociated from the *TREM2* promoter in the mPFC of susceptible mice but not in that of resilient mice (Fig. 3H). The qPCR data revealed the *TREM2* promoter and *TREM2* mRNA levels to have decreased in the mPFC of susceptible mice but not resilient mice (Fig. 3I). Immunoblotting showed decreased Nrf2, TREM2, and arginase 1 protein levels in the mPFC of susceptible mice but not resilient mice (Fig. 3J). Together, these results suggest that the decreased Nrf2 expression, the inhibited TREM2 transcription, and the decreased microglial arginase 1^+^ phenotype in the mPFC are involved in that of susceptible mice but not resilient mice.

### Activation of Nrf2 by SFN increases the arginase 1^+^ microglial phenotype by initiating TREM2 transcription in the mPFC of CSDS mice

**Fig. 4:**
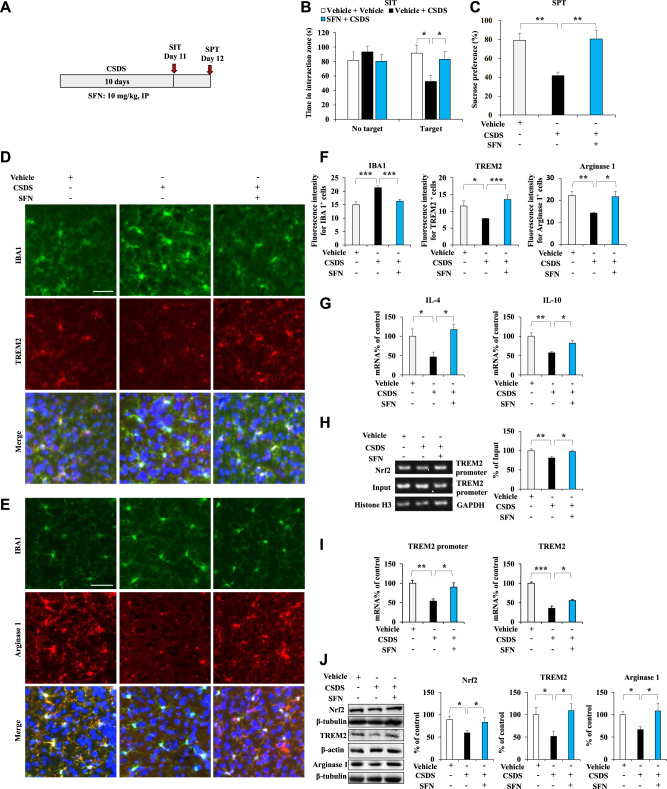
Activation of Nrf2 by SFN induces an arginase 1^+^ microglial phenotype through initiation of TREM2 transcription in the mPFC of SFN-treated CSDS mice. **A** Schedule of behavioral tests and treatments. SFN (10 mg/kg) was injected into mice 30 min before CSDS by intraperitoneal injection. **B** The social interaction test with and without the target (means ± SEMs, *n* = 8 animals per group, one-way ANOVA: *p < 0.05 and ***p < 0.001). **C** The sucrose preference test (means ± SEMs, *n* = 8 animals per group, one-way ANOVA: **p < 0.01). **D**–**F** Representative images and quantification of TREM2, IBA1, and arginase 1 staining in the mPFC. Scale bar = 50 μm (means ± SEMs, *n* = 5 animals per group, one-way ANOVA: **p < 0.01 and ***p < 0.001). **G** qPCR assay showing IL-4 and IL-10 mRNA expression in the mPFC. The data are presented as the means ± SEMs (*n* = 6). *P < 0.05 and **P < 0.01 (one-way ANOVA). **H** ChIP-PCR assay of the TREM2 promoter. Nrf2 protein–DNA cross-linked samples were obtained from the mPFC of control, CSDS, and SFN-treated CSDS mice via coimmunoprecipitation with an anti-Nrf2 antibody. PCR was conducted using TREM2 promoter primers. The data are shown as the mean ± SEM (*n* = 5). *P < 0.05, **P < 0.01 and ***P < 0.001 (one-way ANOVA). **I** qPCR assay for the TREM2 promoter and TREM2 in the mPFC. The data are shown as the mean ± SEM (*n* = 5 or 6). *P < 0.05, **P < 0.01 and ***P < 0.001 (one-way ANOVA). **J** Western blot assay showing Nrf2, TREM2, and arginase 1 protein expression in the mPFC. The data are presented as the means ± SEMs (*n* = 6). *P < 0.05, **P < 0.01 and ***P < 0.001 (one-way ANOVA).

CSDS decreased Nrf2 expression, inhibited TREM2 transcription, and decreased the microglial arginase 1^+^ phenotype in the mPFC of susceptible mice but not resistant mice. Speculating that an Nrf2 activator might correct the expression of these characteristics in susceptible mice, we repeated the administration of SFN (an Nrf2 activator) or vehicle to CSDS mice to further test the hypothesis that the sustained activation of Nrf2 increases the arginase 1^+^ microglial phenotype in the mPFC of CSDS mice by initiating TREM2 transcription. After 10 days of CSDS, the SIT and 1% SPT were performed on Days 11 and 12, respectively (Fig. 4A). In the SIT without a target, the social interaction times were similar among groups; in the SIT with a target, SFN increased social avoidance time in CSDS mice (Fig. 4B). In the SPT, SFN increased sucrose water intake by CSDS mice (Fig. 4C). These results suggested that SFN exerted antidepressant-like effects in the mice. Immunofluorescence staining showed decreased TREM2 immunoreactivity and increased IBA1 immunoreactivity in the mPFC of CSDS mice; these effects were reversed with SFN treatment (Fig. 4D–F). Moreover, immunofluorescence staining revealed decreased arginase 1^+^ microglial immunoreactivity in the mPFC of CSDS mice; these effects were reversed by SFN treatment (Fig. 4E, F). The qPCR results revealed the anti-inflammatory cytokine (IL-4 and IL-10) expression to have decreased in the mPFC of CSDS mice. This decreased expression was reversed with SFN treatment (Fig. 4G). The ChIP-PCR results showed that Nrf2 partially dissociated from the *TREM2* promoter in the mPFC of CSDS mice but not in that of SFN-treated mice (Fig. 4H). The qPCR data showed that the *TREM2* promoter and *TREM2* mRNA levels were decreased in the mPFC of CSDS mice; these effects were reversed with SFN (Fig. 4I). Immunoblotting showed decreased Nrf2, TREM2, and arginase 1 expression in the mPFC of CSDS mice; these changes were also reversed with SFN (Fig. 4J). Thus, sustained activation of Nrf2 by its activator increases the arginase 1^+^ microglial phenotype in the mPFC of CSDS mice, possibly by initiating TREM2 transcription.

### Knockout of Nrf2 decreases TREM2 and arginase 1^+^ microglial phenotypes in the mPFC of Nrf2 KO mice

**Fig. 5:**
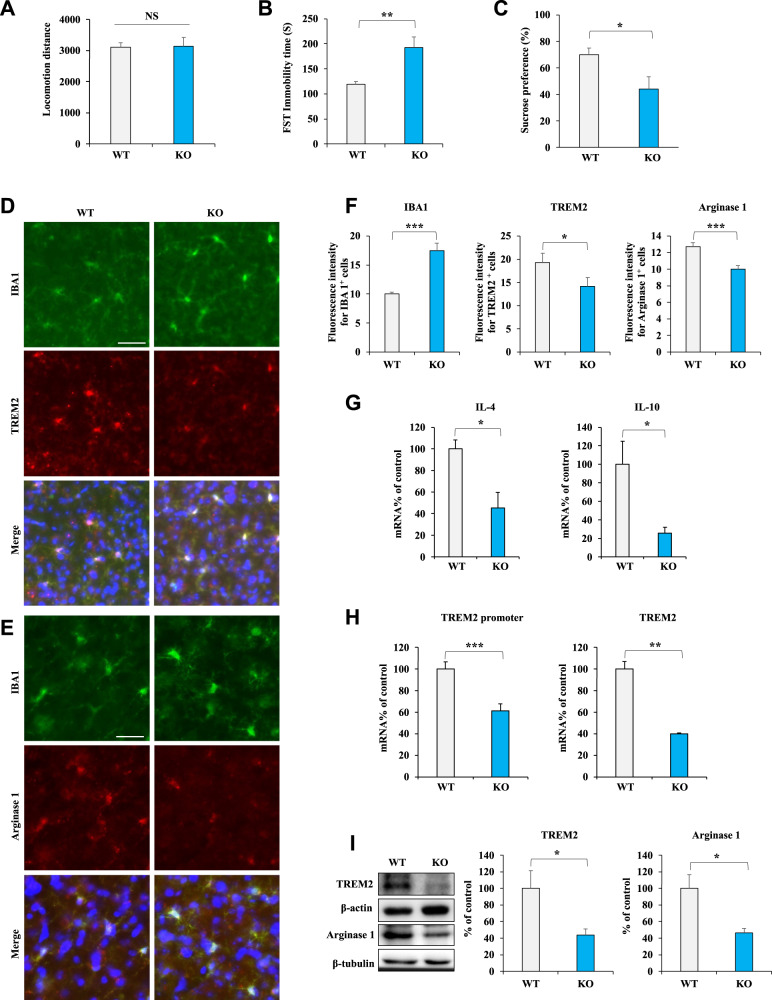
Knockout of Nrf2 decreases TREM2 and the microglial arginase 1^+^ phenotype in the mPFC of Nrf2 KO mice. **A** The locomotion test. **B** FST: forced swimming test. **C** SPT: sucrose preference test (mean ± SEM, *n* = 7 or 8 per group, Student’s *t* test). **D**–**F** Representative images and quantification of TREM2, IBA1, and arginase 1 staining in the mPFC. Scale bar = 50 μm (mean ± SEM, *n* = 5 per group, Student’s *t* test). **G** qPCR assay for IL-4 and IL-10 in the mPFC. The data are shown as the mean ± SEM (*n* = 5 or 6). *P < 0.05 and ***P < 0.001 (Student’s *t* test). **H** qPCR assay for the TREM2 promoter and TREM2 in the mPFC. The data are shown as the mean ± SEM (*n* = 6). *P < 0.05 and ***P < 0.001 (Student’s *t* test). **I** Western blot assay for TREM2 and arginase 1 protein expression in the mPFC. The data are shown as the mean ± SEM (*n* = 5 or 6). *P < 0.05 and **P < 0.01 (Student’s *t* test).

We investigated whether downregulating Nrf2 can decrease TREM2 and arginase 1^+^ microglial phenotypes. We employed Nrf2 KO mice and wild-type (WT) mice. The behavior test revealed depression-like behavior in the Nrf2 KO mice (Fig. 5A–C). This finding is consistent with previous reports [28, 36, 37]. There were no differences in locomotion between the two groups (Fig. 5A). Regarding the FST, the immobility times increased for Nrf2 KO mice (Fig. 5B). Furthermore, a diminished preference for sucrose was observed in Nrf2 KO mice (Fig. 5C). Immunofluorescence staining with anti-TREM2 and anti-IBA1 antibodies in the mPFC sections revealed decreased TREM2 immunoreactivity and increased IBA1 immunoreactivity in the Nrf2 KO mice (Fig. 5D–F). In addition, the colocalization of arginase 1 and IBA1 was prominently observed in the mPFC (Fig. 5E). The data showed that arginase 1^+^ microglial immunoreactivity decreased in the mPFC of Nrf2 KO mice (Figs. 4E and 4F). The qPCR revealed decreased anti-inflammatory cytokine (IL-4 and IL-10) expression in the mPFC of Nrf2 KO mice (Fig. 5G). To further validate the connections concerning the regulation of TREM2 in vivo, we examined the *TREM2* promoter and *TREM2* mRNA levels in the mPFC of Nrf2 KO and WT mice. The qPCR results revealed decreased *TREM2* promoter and *TREM2* mRNA levels in the mPFC of Nrf2 KO mice (Fig. 5H). Immunoblotting showed decreased TREM2 and arginase 1 protein levels in the mPFC of Nrf2 KO mice (Fig. 5I). These findings indicate that the knockout of Nrf2 decreases TREM2 and arginase 1^+^ microglial phenotypes in the mPFC of Nrf2 KO mice with depression-like behavior.

### Downregulation of TREM2 decreases the arginase 1^+^ microglial phenotype in the mPFC of SFN-treated CSDS mice

**Fig. 6:**
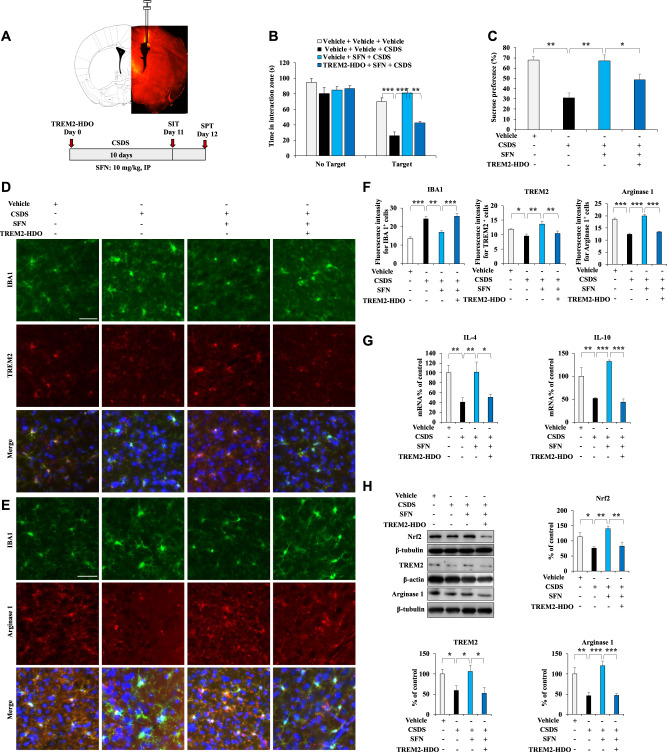
Downregulation of TREM2 attenuates the microglial arginase 1^+^ phenotype in the mPFC of SFN-treated CSDS mice. **A** Graphical illustration of the intracerebroventricular injection site and the distribution of CY5-TREM2-HDO in the mouse brain. Schedule of behavioral tests and treatments. CY5-TREM2-HDO or TREM2-HDO was administered to the mice on Day 0 by intracerebroventricular injection. SFN (10 mg/kg) was intraperitoneally injected into mice 30 min before CSDS. **B** The social interaction test with and without the target (means ± SEMs, *n* = 7 or 9 animals per group, one-way ANOVA: *p < 0.05 and ***p < 0.001). **C** The sucrose preference test (means ± SEMs, *n* = 7 or 9 animals per group, one-way ANOVA: **p < 0.01). **D**–**F** Representative images and quantification of TREM2, IBA1, and arginase 1 staining in the mPFC. Scale bar = 50 μm (means ± SEMs, *n* = 4 animals per group, one-way ANOVA: **p < 0.01 and ***p < 0.001). **G** qPCR assay showing IL-4 and IL-10 mRNA expression in the mPFC. The data are presented as the means ± SEMs (*n* = 6). *P < 0.05, **P < 0.01 and ***P < 0.001 (one-way ANOVA). **H** Western blot assay showing Nrf2, TREM2, and arginase 1 protein expression in the mPFC. The data are presented as the means ± SEMs (*n* = 6). *P < 0.05, **P < 0.01 and ***P < 0.001 (one-way ANOVA).

We downregulated TREM2 in the brains of SFN-treated CSDS mice by treating them with TREM2-HDO to assess whether Nrf2 activation by SFN increases the arginase 1^+^ microglial phenotype in the mPFC of SFN-treated CSDS mice according to TREM2 expression. An i.c.v. injection of TREM2-HDO significantly decreased TREM2 expression in the mPFC of adult mice (Fig. S4), indicating that TREM2-HDO efficiently silenced TREM2 gene expression. A single i.c.v. injection of TREM2-HDO (200 nM, 2 μl) significantly attenuated the antidepressant-like effects of SFN (Fig. 5A–C). In the SIT without a target, the social interaction times were similar among groups, while in the SIT with a target, TREM2-HDO significantly attenuated the increased social avoidance time in SFN-treated CSDS mice (Fig. 6B). In the SPT, TREM2-HDO significantly attenuated the enhanced sucrose water intake in SFN-treated CSDS mice (Fig. 6C). The results suggest that TREM2-HDO attenuated SFN-induced antidepressant-like effects in CSDS mice. Immunofluorescence staining showed that TREM2-HDO significantly attenuated the beneficial effects of SFN on TREM2 immunoreactivity and IBA1 immunoreactivity in the mPFC of SFN-treated CSDS mice (Fig. 6 D–F). Moreover, TREM2-HDO significantly attenuated the beneficial effects of SFN on arginase 1^+^ microglial immunoreactivity in the mPFC of SFN-treated CSDS mice (Fig. 6E, F). The qPCR results showed that TREM2-HDO significantly attenuated the beneficial effects of SFN on anti-inflammatory cytokine (IL-4 and IL-10) expression in the mPFC of CSDS mice (Fig. 6G). Immunoblotting showed that TREM2-HDO significantly attenuated the beneficial effects of SFN on the expressions of Nrf2, TREM2, and arginase 1 in the mPFC of CSDS mice (Fig. 6H). Taken together, these results suggest that Nrf2 activation by SFN increases the arginase 1^+^ microglial phenotype in the mPFC of SFN-treated CSDS mice according to TREM2 expression.

### TREM2 regulates the microglial arginase 1^+^ phenotype and is associated with the BDNF-TrkB signaling pathway

**Fig. 7:**
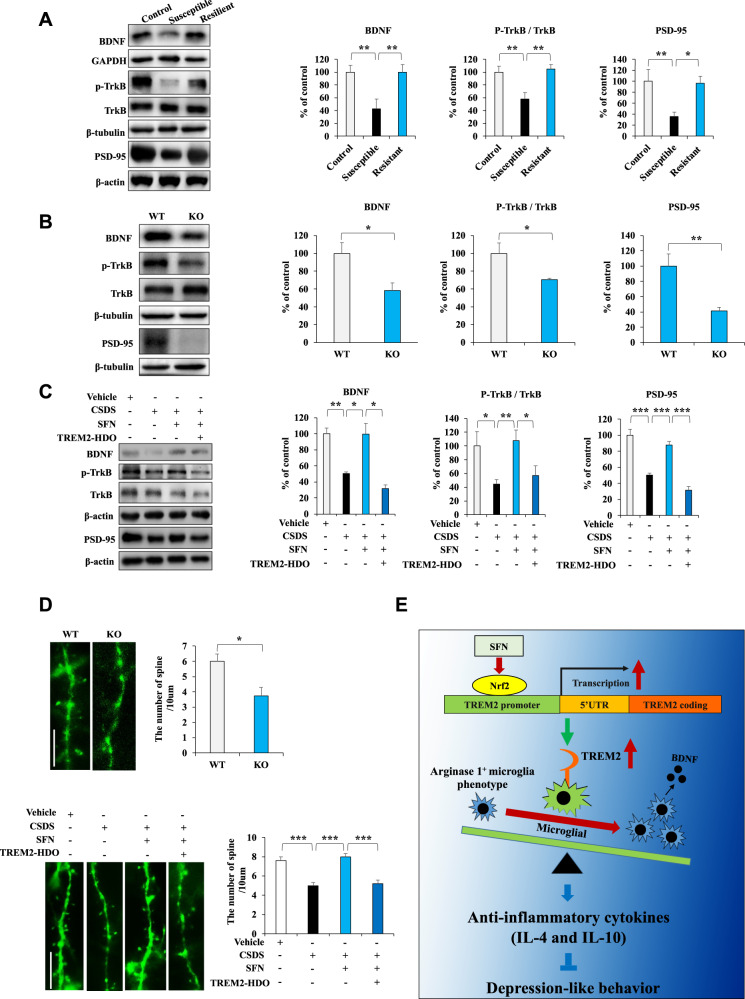
TREM2 regulates the arginase 1^+^ microglial phenotype and is associated with the BDNF-TrkB signaling pathway. **A**–**C** Western blot assay showing BDNF, PSD-95, and the ratio of p-TrkB/TrkB expression in the mPFC. The data are presented as the means ± SEMs (*n* = 5 or 6). *P < 0.05, **P < 0.01, and ***P < 0.001 (one-way ANOVA). **D** Representative photomicrographs and quantification of the dendritic spine density in the mPFC. Scale bar = 10 μm. The data are presented as the means ± SEMs (*n* = 5). *P < 0.05 and ***P < 0.001 (one-way ANOVA). **E** The working model for Nrf2-induced TREM2 transcription in the model of depression. Activation of Nrf2 by SFN induces an arginase 1^+^ microglial phenotype by initiating TREM2 transcription and thus ameliorates depression-like behavior in mice.

Multiple lines of evidence suggest that the BDNF-TrkB signaling pathway plays a role in the pathophysiology of depression and the therapeutic mechanisms of antidepressants [38–46]. The activation of the anti-inflammatory phenotype of microglia reportedly coordinates the expression of various anti-inflammatory factors and increases BDNF expression, effecting stress resilience in mice [29, 47]. Immunoblotting suggested that the levels of BDNF decreased in LPS-treated primary microglia: an outcome reversed with SFN (Fig. S5). We further observed that TREM2-HDO attenuated the increased BDNF expression in LPS + SFN-treated primary microglia (Fig. S5). Next, we investigated the BDNF-TrkB signaling pathway in vivo. Immunoblotting showed that the levels of BDNF, the p-TrkB/TrkB ratio, and postsynaptic density protein 95 (PSD-95) expression had decreased in the mPFC of susceptible mice and Nrf2 KO mice, whereas the levels of these proteins had increased in the mPFC of resistant mice (Fig. 7A, B). Moreover, TREM2-HDO significantly attenuated the beneficial effects of SFN on alterations in the p-TrkB/TrkB ratio and expressions of BDNF and PSD-95 in the mPFC of SFN-treated CSDS mice (Fig. 7C). Using Nrf2 KO Thy1-YFP and Thy1-YFP mice, we observed a significantly lower dendritic spine density in the mPFC of Nrf2 KO and CSDS mice than in control mice. We further found that SFN significantly ameliorated the reduced dendritic spine density in the mPFC of CSDS mice (Fig. 7D). The beneficial effects of SFN on the dendritic spine density in the mPFC of SFN-treated CSDS mice were significantly inhibited by a single i.c.v. injection of TREM2-HDO (Fig. 7D). These results suggest that the knockout Nrf2 and downregulation of TREM2 expression decrease the arginase 1^+^ microglial phenotype in the mPFC of Nrf2 KO mice and that SFN-induced changes in CSDS mice are associated with the BDNF-TrkB signaling pathway.

## Discussion

The present study shows that Nrf2 regulates TREM2 transcription. Nrf2 activation by SFN induced an anti-inflammatory microglial phenotype by increasing TREM2 expression in LPS-treated primary microglia. Activation of Nrf2 by SFN increased the microglial arginase 1^+^ phenotype by initiating TREM2 transcription in the mPFC of CSDS mice. The knockout of Nrf2 decreased TREM2 and arginase 1^+^ microglial phenotypes in the mPFC of Nrf2 KO mice with depression-like behavior. Downregulating TREM2 expression decreased the microglial arginase 1^+^ phenotype in the mPFC of SFN-treated CSDS mice. Finally, the knockout of Nrf2 and downregulation of TREM2 expression decreased the microglial arginase 1^+^ phenotype in the mPFC of Nrf2 KO mice and that SFN-induced effects in CSDS mice were associated with the BDNF-TrkB signaling pathway.

Microglia exhibit phenotypic plasticity that is stimulated by different cytokines to regulate physiological responses and behavioral outcomes during stress [47, 48]. Microglia adopt proinflammatory and anti-inflammatory phenotypes according to the microenvironment [4–6]. Activation of the anti-inflammatory phenotype of microglia through the regulated release of various anti-inflammatory (IL-4 and IL-10) and neurotrophic factors effects neuronal protection [4, 7–9]. TREM2 is a cell surface receptor of the lectin-like immunoglobulin superfamily that is expressed at high levels in microglia and functions as a key regulator of the inflammatory response [10]. Further, TREM2 plays an essential role in maintaining immune homeostasis in the brain by promoting tissue debris clearance and resolution of latent inflammatory responses [13]. Activation of TREM2 stimulates phagocytic activity in microglia and downregulates the expression of TNF-α and inducible nitric oxide synthase (iNOS) [49]. While opposing effects were observed following TREM2 overexpression, TREM2 deficiency attenuated the phagocytic activities of microglia and exacerbated ischemic damage [50]. Thus, TREM2 is simultaneously an anti-inflammatory receptor and promotor of phagocytic activity. These results indicate that TREM2 might play an important role in controlling microglial proinflammatory and anti-inflammatory phenotypes. The specific knockdown of TREM2 in microglia from the LHb reportedly induces proinflammatory cytokine expression and microglial activation, resulting in depression-like behaviors in mice [15]. However, the role of TREM2 in the pathogenesis of depression had not been comprehensively assessed.

The present study provides, to the best of our knowledge, the first direct evidence that Nrf2 regulates TREM2 transcription to stimulate TREM2 mRNA and protein expression. Activation of Nrf2 by SFN induced an anti-inflammatory microglial phenotype by increasing TREM2 expression in LPS-treated primary microglia. In addition, activation of Nrf2 by SFN increased the microglial arginase 1^+^ phenotype by initiating TREM2 transcription in the mPFC of CSDS mice. Arginase 1 is a marker of the anti-inflammatory phenotype of microglia [5, 6]. Further, the activation of the anti-inflammatory phenotype of microglia regulates the release of various anti-inflammatory factors and increases neurotrophic factor release, enhancing neuronal protection [4–7]. Our results show that TREM2-HDO significantly attenuates the beneficial effects of SFN on anti-inflammatory cytokine (IL-4 and IL-10) expression and increases the microglial arginase 1^+^ phenotype in the mPFC of CSDS mice. Therefore, activation of Nrf2 by SFN increases the anti-inflammatory microglial phenotype in the mPFC of SFN-treated CSDS mice according to TREM2 expression, resulting in a neuroprotective effect (Fig. 7E). This study also found that the downregulation of TREM2 decreases Nrf2 expression and that TREM2 overexpression upregulates Nrf2 expression in BV2 cells. These activities may constitute a positive feedback loop for the Nrf2 and TREM2 interaction.

Based on accumulating evidence, the BDNF-TrkB signaling pathway plays a key role in the pathophysiology of depression and the therapeutic mechanisms of antidepressants [38–46]. Several studies have shown that decreased BDNF levels and polymorphisms in the BDNF gene are associated with MDD. Low plasma BDNF levels have been associated with suicidal behaviors in patients with major depression [51]. In addition, reduced BDNF levels were detected in the parietal cortex, mPFC, and hippocampus of the postmortem brains of patients with psychiatric disorders, including MDD [52, 53]. In contrast, the parietal cortex of postmortem tissues of patients with MDD who received antidepressant treatment exhibited higher levels of BDNF than did untreated patients with MDD [54]. The activation of the anti-inflammatory phenotype of microglia reportedly coordinates with the expression of various anti-inflammatory factors and increases BDNF expression, effecting stress resilience in mice [29, 47]. In the current study, we noticed that the activation of Nrf2 by SFN induces the microglial arginase 1^+^ phenotype, activates the BDNF-TrkB signaling pathway, and reverses the reduced dendritic spine density in the mPFC of CSDS mice through initiation of TREM2 transcription. Therefore, our findings suggest that Nrf2 may promote TREM2 transcription in microglia with an anti-inflammatory phenotype, resulting in BDNF upregulation (Fig. 7E). BDNF, through activation of the TrkB signaling pathway, reverses the reduced dendritic spine density in the mPFC of CSDS mice and promotes antidepressant-like effects. Moreover, we have previously reported that Nrf2 was downregulated to a greater extent in the parietal cortex of MDD patients than in control subjects [55]. The present study suggests that Nrf2 could regulate TREM2 transcription. Therefore, TREM2 levels in the parietal cortex of patients with MDD warrants further examination. Taken together, the inhibition of Nrf2-induced TREM2 transcription may contribute to CSDS-induced depression-like behaviors, and the activation of Nrf2-induced TREM2 transcription may confer antidepressant-like effects.

As for the limitation of the present study, we did not explore the role of microglia-derived BDNF in the pathogenesis of depression and whether microglia-derived BDNF plays a dominant role in the pathogenesis of depression. Future detailed studies on these topics are needed. Further, the mechanism by which TREM2 regulates microglial BDNF remains vague and will require more delineation. Nrf2 can bind to the BDNF exon I promoter, resulting in BDNF transcription [28]. We found that the downregulation of TREM2 decreases Nrf2 expression and that the overexpression of TREM2 increases Nrf2 expression in BV2 cells. Therefore, TREM2 may regulate BDNF expression through Nrf2.

In conclusion, our study demonstrated that Nrf2 regulates TREM2 transcription by binding to its promoter. Further, the inhibition of Nrf2-induced TREM2 transcription may play a role in the pathophysiology of depression. Finally, activation of Nrf2-induced TREM2 transcription may attenuate depression-like behaviors in CSDS mice. The present study thus suggests that alterations in the interaction between Nrf2 and TREM2 may contribute to the pathogenesis of depression in the CSDS mouse model.

## Supplementary information

Supplement material and figures

## Data Availability

The datasets used and/or analyzed during the current study are available from the corresponding author on reasonable request.
