# Supplementary material for: Nrf2 regulates the arginase 1+ microglia phenotype through the initiation of TREM2 transcription, ameliorating depression-like behavior in mice
Source: Transl Psychiatry. 2022 Oct 31;12:459. doi: 10.1038/s41398-022-02227-y (PMC9622811; doi:10.1038/s41398-022-02227-y)
Supplement: Supplementary file 1 — Supplement material and figures [file 41398_2022_2227_MOESM1_ESM.docx]

**Supplemental information**

**Chronic social defeat stress (CSDS) model, social interaction test (SIT), locomotion, forced swimming test (FST) and 1% sucrose preference test (SPT)**

The CSDS procedure was performed as previously reported ^1-4^. C57BL/6 mice or Thy1-YFP mice were defeated by different CD1 mice for 10 min for a total of 10 days. Following the social defeat session, CD1 mice and C57BL/6 mice or Thy1-YFP mice were housed in half of the cage for 24 h using a perforated Plexiglas divider, which allowed visual, olfactory, and auditory contacts over 24 h. C57BL/6 mice or Thy1-YFP mice were raised separately after the end of the last session. The social interaction test was performed to examine the mice that were accordingly susceptible and resistant.

For the SIT, an open box (42 × 42 cm) with an interaction zone that included a mesh-plastic target box (10 × 4.5 cm) and two opposing corner zones was employed. The two parts were used for this test (no social or social targets). For the no social target, the test mouse was placed into an open field arena for 2.5 min with no social target (no CD1 mouse) in the mesh-plastic target box. After the no social target test, the mouse was placed into the open field arena again in the second 2.5 min with a social target (a novel CD1 mouse) in the mesh-plastic target box. The residence time in the interaction zone was counted using the stopwatch, and the time of ratio for social target, and no social target was calculated accordingly. Approximately 70% of mice were susceptible to social defeat stress.

Locomotion: the locomotor activities of mice were analyzed by using Ethovision XT 14.0 software (Noldus). The cumulative exercise was recorded in 60 minutes. FST: The mice were placed individually in a cylinder (diameter: 23 cm; height: 31 cm) containing 15 cm of water, maintained at 23 ± 1 °C. Mice were monitored using a video tracking system (Ethovision XT 14.0) for 6 minutes. For the SPT, mice were habituated to a 1% sucrose solution for 48 h before the test day, and then the mice were deprived of water and food for 4 h followed by a preference test with water and 1% sucrose for 1 h. Bottles containing water and sucrose were weighed before and at the end of this period to calculate the sucrose preference (%) ^5-7^.

**Luciferase reporter assay**

The luciferase reporter assay was performed as described previously ^8, 9^. BV2 cells were transfected with pRL-TK Renilla luciferase plasmid and differently lengths of *Trem2* promoter luciferase reporter plasmid in 6-wells plates, followed by treatment with SFN or siRNA-Nrf2. Following transfection for 24 h, the cells were collected and subjected to analysis using a dual-luciferase reporter assay kit (Promega, Madison, USA) according to the manufacturer’s instructions.

**Chromatin immunoprecipitation (ChIP) assay**

ChIP was performed as described previously ^8, 9^. Cells or brain samples were subjected to ChIP assay according to the manual of the SimpleChIP® Enzymatic Chromatin IP Kit (Cell Signaling). Specifically, 7.5 μg of Nrf2 antibody (Abcam) was added to the homogenate of the cell or brain lysates. The mixture was incubated overnight at 4 °C. The washing, elution, and reverse cross-linking of free DNA were performed according to the manufacturer’s protocol. *Trem2* promoter-specific primers were used to amplify the promoter region. The primer sequences were: forward 5’ CCAGACCCCAGTCCTGACTATT 3’; reverse 5’ TTGTGCA AGATCTCGTC TTTCC’. The PCR amplicons were separated on a 2% agarose gel after 35 cycles of PCR (denaturation at 95 °C for 30 s, annealing at 58 °C for 30 s, and extension at 72 °C for 30 s).

**Quantitative PCR (qPCR) assay**

Levels of *Trem2* promoter mRNA and *Trem2* mRNA were analyzed by quantitative real-time PCR. RNA was extracted by using Eastep® Super Kit (Promega), and then reverse transcription was performed with GoScript^TM^ Reverse Transcriptase Mix, Oligo (dT) (Promega). All qPCR reactions were performed by using the 788BR05175 Real-Time PCR System and ChamQ^TM^ SYBR® qPCR Master Mix Kit (Vazyme). The target gene expression was calculated as 2−ΔΔCt method. Forty cycles of PCR amplification were performed as follows: denature at 95°C for 30 s, anneal at 55°C for 30 s, and extend for 30 s at 72°C. The primer sequences were: *Trem2* promoter forward 5’ CCAGACCCCAGTCCTGACTATT 3’; reverse 5’ TTGTGCAAGATCTCGTC TTTCC’; *Trem2* forward 5’ ATGGGACCTCTCCACCAGTT 3’; reverse 5’ GGGTCCAGTGAGGATCTGAA’; *IL-4* forward 5’ CCTGCTCTTCTTTCTCGAATGT 3’; reverse 5’ TTTCAGTGATGTGGACTTGGAC’, and *IL-10* forward 5’ AGGCGCTGTCATCGATTTCTC 3’; reverse 5’ TGCTCCACTGCCTTGCTCTTA’.

**Immunofluorescence staining**

Mice were anesthetized with sodium pentobarbital and perfused transcardially with 10 mL of isotonic saline, followed by 40 mL of ice-cold 4% paraformaldehyde in 0.1 M phosphate buffer (pH 7.4). Brain samples were collected after perfusion and post-fixed overnight at 4 °C. Serial coronal sections (50 μm) of brain tissue were cut in ice-cold, 0.01 M phosphate-buffered saline (pH 7.5) using a vibrating blade microtome (VT1000S, Leica Microsystems AG, Wetzlar, Germany). For staining, the cells or mouse brain sections were incubated with 3% hydrogen peroxide at room temperature for 10 min after fixation with 4% paraformaldehyde. Then, the sections were blocked with blocking solution for 1 h and incubated with primary antibodies (anti-TREM2, 1:500, ab86491, abcam; anti-IBA1, 1:500, ab283319, abcam; anti-arginase1, 1:500, 93668S, Cell Signaling Technology) 48 hours. The third day, Alexa Fluor 488- or 568- conjugated isotype-specific secondary antibodies were incubated for 1 h at room temperature. Images were collected using an Olympus fluorescence microscope (Olympus BX53, Tokyo, Japan). The fluorescence intensity was quantified in the anterior regions (0.018 mm^2^) of each brain section using Image J.

**Immunoblotting assay**

Cells or brain samples were lysed in RIPA buffer (20 mM pH 7.5 Tris-HCl, 150 mM NaCl, 1 mM Na_2_EDTA, 1 mM EGTA, 1% Triton, 2.5 mM sodium pyrophosphate, 1 mM beta-glycerophosphate, 1 mM Na_3_VO_4_, 1 μg/ml leupeptin, 1 mM phenylmethylsulfonyl fluoride). The concentrations of total proteins were examined by Bradford assay. 30 μg Proteins were resolved on 7.5%, 10%, or 15% polyacrylamide gels, according to each marker’s molecular weight, and then transferred to polyvinylidenedifluoride (PVDF) membrane. For the immunodetection, the blots were blocked with 2% BSA plus 5% nonfat dry milk in TBST (TBS + 0.1% Tween-20) for 1 h at room temperature (RT), and then incubated with primary antibodies (The concentration is selected with the manufacturer’s instructions) overnight at 4°C. Next day, blots were washed three times in TBST and incubated with horseradish peroxidase conjugated anti-rabbit antibody (1:5000) or anti-mouse antibody (1:5000) for 1hour, at RT. After the three times washes with TBST, the bands were detected by using enhanced chemiluminescence (ECL) detection reagents (GE Healthcare) and exposed to Tanon-5200CE imaging system (Tanon, Shanghai, China). The quantification was carried out with ImageJ software. The primary antibodies used in assays were listed below: TREM2 antibody (ab86491), Nrf2 antibody (ab137550), and BDNF antibody (ab108319) was purchased from abcam, arginase1 antibody (66129-1-Ig) was purchased from proteintech , phospho-TrkB antibody (4621S), PSD-95 antibody (7E3) and TrkB antibody (4603S) was purchased from Cell Signaling Technology, β-actin antibody（AF7018）β-tubulin antibody ([AF7011](http://www.affbiotech.cn/goods-6286-AF7011-Tubulin_beta_Antibody.html)) and GAPDH antibody ([AF7021](http://www.affbiotech.cn/goods-6289-AF7021-GAPDH_Antibody.html)) was purchased from Affinity. The HRP-conjugated anti-rabbit IgG antibody, and anti-mouse IgG antibody were purchased from BIO-RAD.

**Dendritic spine analysis**

CSDS of Thy1-YFP mice was performed for 10 days (day 1 – day 10). After the social interaction test on day 11, CSDS susceptible mice were selected. TREM2-HDO was injected i.c.v. to mice on day 0, and SFN was injected i.p. to mice 30 min before the CSDS. On the day 13, the CSDS of Thy1-YFP mice or Nrf2 KO Thy1-YFP mice were deeply anesthetized with sodium pentobarbital and perfused transcardially with 10 ml of isotonic saline, followed by 40 ml of ice-cold 4% paraformaldehyde in 0.1-M phosphate buffer (pH 7.4). Brains were removed from the skulls and postfixed overnight at 4°C with the same fixative. For dendritic spine analysis, 50-μm thick serial coronal sections of brain tissue were cut in ice-cold, 0.01-M phosphate-buffered saline (pH 7.5) using a vibrating blade microtome (VT1000S, Leica Microsystems AG, Wetzlar, Germany). The sections were mounted on gelatinized slides, dehydrated, cleared, and coverslipped under Permount^®^ (Fisher Scientific, Fair Lawn, NJ, USA). Next, sections were observed in a fluorescent microscope (Olympus BX53, Japan), and pictures were then taken and dendritic spine was quantified in 10 μm of each dendritic in a blinded manner.

**Supplemental figure**

**Fig S1. The information for series of luciferase-conjugated constructs of human TREM2 promoters with predicted Nrf2 binding sites**

**Fig S2. The information for site 3 of human TREM2 promoters and corresponding mouse TREM2 promoter**

**Fig S3. TREM2 influences Nrf2 expression**

**A**: qPCR analysis of TREM2 and Nrf2 mRNA expression in BV2 cells after the administration of different doses of TREM2-HDO treatment. The data are presented as the means ± SEM (n = 4). **P* < 0.05 and ***P* < 0.01 (one-way ANOVA). **B**: Western blot assay showing TREM2 and Nrf2 protein expression in BV2 cells after the administration of different doses of TREM2-HDO treatment. The data are presented as the means ± SEM (n = 4). ***P* < 0.01 and ****P* < 0.001 (one-way ANOVA). **C**: qPCR analysis of TREM2 and Nrf2 mRNA expression in BV2 cells after transfection with different doses of GST-TREM2. The data are presented as the means ± SEM (n = 4). **P* < 0.05, ***P* < 0.01, and ****P* < 0.001 (one-way ANOVA). **D**: Western blot assay showing TREM2 and Nrf2 protein expression in BV2 cells after transfection with different doses of GST-TREM2. The data are presented as the means ± SEM (n = 4). **P* < 0.05, ***P* < 0.01, and ****P* < 0.001 (one-way ANOVA).

**Fig S4.** **TREM2-HDO decreases TREM2 protein expression**

Representative images for TREM2 western blot and quantification analysis of TREM2 in the mPFC of TREM2-HDO-treated mice. The data are the mean ± SEM (n = 4). **P* < 0.05 (Student t-test).

**Fig S5. The levels of BDNF protein expression in LPS-treated primary microglia after SFN administration**

Representative images for BDNF western blot and quantification analysis of BDNF in LPS-treated primary microglia after SFN administration. The data are the mean ± SEM (n = 5 or 6). **P* < 0.05 (one-way ANOVA).

**Reference**

1. Berton O, McClung CA, Dileone RJ, Krishnan V, Renthal W, Russo SJ *et al.* Essential role of BDNF in the mesolimbic dopamine pathway in social defeat stress. *Science* 2006; **311**(5762)**:** 864-868.

2. Golden SA, Covington HE, 3rd, Berton O, Russo SJ. A standardized protocol for repeated social defeat stress in mice. *Nature protocols* 2011; **6**(8)**:** 1183-1191.

3. Tsankova NM, Berton O, Renthal W, Kumar A, Neve RL, Nestler EJ. Sustained hippocampal chromatin regulation in a mouse model of depression and antidepressant action. *Nat Neurosci* 2006; **9**(4)**:** 519-525.

4. Zhao T, Huang GB, Muna SS, Bagalkot TR, Jin HM, Chae HJ *et al.* Effects of chronic social defeat stress on behavior and choline acetyltransferase, 78-kDa glucose-regulated protein, and CCAAT/enhancer-binding protein (C/EBP) homologous protein in adult mice. *Psychopharmacology (Berl)* 2013; **228**(2)**:** 217-230.

5. Zhang JC, Wu J, Fujita Y, Yao W, Ren Q, Yang C *et al.* Antidepressant effects of TrkB ligands on depression-like behavior and dendritic changes in mice after inflammation. *Int J Neuropsychopharmacol* 2014; **18**(4)**:** pyu077.

6. Zhang JC, Yao W, Dong C, Yang C, Ren Q, Ma M *et al.* Comparison of ketamine, 7,8-dihydroxyflavone, and ANA-12 antidepressant effects in the social defeat stress model of depression. *Psychopharmacology (Berl)* 2015; **232**(23)**:** 4325-4335.

7. Zhang JC, Yao W, Dong C, Yang C, Ren Q, Ma M *et al.* Prophylactic effects of sulforaphane on depression-like behavior and dendritic changes in mice after inflammation. *The Journal of nutritional biochemistry* 2017; **39:** 134-144.

8. Wang ZH, Gong K, Liu X, Zhang Z, Sun X, Wei ZZ *et al.* C/EBPbeta regulates delta-secretase expression and mediates pathogenesis in mouse models of Alzheimer's disease. *Nature communications* 2018; **9**(1)**:** 1784.

9. Wu Z, Xia Y, Wang Z, Su Kang S, Lei K, Liu X *et al.* C/EBPbeta/delta-secretase signaling mediates Parkinson's disease pathogenesis via regulating transcription and proteolytic cleavage of alpha-synuclein and MAOB. *Mol Psychiatry* 2020; **26**(2):568-585.
